# Dating Violence, Sexual Violence, and Bullying Victimization Among High School Students —Youth Risk Behavior Survey, United States, 2021

**DOI:** 10.15585/mmwr.su7201a8

**Published:** 2023-04-28

**Authors:** Heather B. Clayton, Greta Kilmer, Sarah DeGue, Lianne F. Estefan, Vi D. Le, Nicolas A. Suarez, Bridget H. Lyons, Jemekia E. Thornton

**Affiliations:** ^1^Division of Violence Prevention, National Center for Injury Prevention and Control, CDC; ^2^Division of Adolescent and School Health, National Center for HIV, Viral Hepatitis, STD, and TB Prevention, CDC

## Abstract

Experiences of teen dating violence (TDV), sexual violence, and bullying during adolescence are all forms of interpersonal violence victimization (IVV) and are associated with health and behavioral issues during adulthood. Data from the nationally representative 2011–2021 Youth Risk Behavior Surveys were used to estimate the 2021 prevalence of IVV reported by U.S. high school students. IVV included past-year sexual TDV, physical TDV, sexual violence by anyone, electronic bullying, being bullied on school property, and lifetime forced sex and was analyzed by demographic characteristics and sex of sexual contacts. This report also explored trends in IVV over this 10-year period among U.S. high school students. In 2021, a total of 8.5% of students reported physical TDV, 9.7% reported sexual TDV, 11.0% reported sexual violence by anyone (with 59.5% of those also reporting sexual TDV), 15.0% reported bullying on school property, and 15.9% reported electronic bullying victimization during the past 12 months; 8.5% also reported experiencing forced sex in their lifetime. Disparities were observed for each form of IVV assessed for females and for most forms of IVV among racial and ethnic minority students; students who identified as lesbian, gay, bisexual, questioning, or other (LGBQ+); and students who reported their sexual contacts as same sex only or both sexes. Trend analyses indicated that physical TDV, sexual TDV, any physical or sexual TDV, and both physical and sexual TDV victimization decreased from 2013 to 2021 (although sexual TDV increased from 2019 to 2021). Any bullying victimization decreased from 2011 to 2021. Lifetime forced sexual intercourse decreased from 2011 to 2015, then increased from 2015 to 2021. Being bullied on school property was unchanged from 2011 to 2017, then decreased from 2017 to 2021. Sexual violence by anyone increased from 2017 to 2021. This report highlights disparities in IVV and provides the first national estimates among Native Hawaiian or other Pacific Islander youths. Findings, including trend analyses indicating recent increases in certain forms of IVV, point to the continued urgency of violence prevention efforts for all U.S. youths and especially those who are disproportionately affected by IVV.

## Introduction

Teen dating violence (TDV), sexual violence, and bullying during adolescence, all forms of interpersonal violence victimization (IVV), are associated with later revictimization, substance use, physical and mental health issues, and suicidal ideation (*1*). The most recent available data from the 2021 nationally representative Adolescent Behaviors and Experiences Study (ABES), designed to capture adolescent experiences during the COVID-19 pandemic, found that 9.6% of high school students reported experiencing any sexual violence, 7.7% experienced sexual TDV, 6.4% experienced physical TDV, 13.8% experienced electronic bullying, and 12.5% were bullied at school during the year before the survey (https://www.cdc.gov/healthyyouth/data/abes/tables/summary.htm#UIV). In addition, 6.7% of students from the 2021 ABES reported lifetime experience of forced sexual intercourse. Substantial disparities exist in the prevalence of IVV. Females, racial and ethnic minority populations, and sexual minority youths experienced disproportionately greater prevalence of these forms of IVV (*1*,*2*). Understanding the pattern of disparities in IVV is important for developing prevention and intervention efforts.

Using data from the national Youth Risk Behavior Survey (YRBS), this report presents 2021 prevalence estimates for TDV, sexual violence, and bullying victimization of U.S. high school students by sex, race and ethnicity, sexual identity, and sex of sexual contacts. In addition, this report presents 2011–2021 trends for TDV, sexual violence, and bullying victimization among U.S. high school students and compares 2019 with 2021 data to explore potential differences in past-year estimates before (fall 2019) and during (fall 2021) the COVID-19 pandemic. These findings can be used when developing prevention and intervention efforts to address health inequities and improve long-term behavioral and health outcomes of U.S. youths.

## Methods

### Data Source

This report includes data from the 2021 YRBS (N = 17,232), a cross-sectional, school-based survey conducted biennially since 1991. Each survey year, CDC collects data from a nationally representative sample of public and private school students in grades 9–12 in the 50 U.S. states and the District of Columbia. Additional information about YRBS sampling, data collection, response rates, and processing is available in the overview report of this supplement (*3*). The prevalence estimates for types of IVV for the overall study population and by sex, race and ethnicity, grade, and sexual identity are available at https://nccd.cdc.gov/youthonline/App/Default.aspx. The full YRBS questionnaire, data sets, and documentation are available at https://www.cdc.gov/healthyyouth/data/yrbs/index.htm. This activity was reviewed by CDC and was conducted consistent with applicable federal law and CDC policy.*

### Measures

This analysis included six standard measures of IVV and three composite variables created from those measures (Table 1). The standard measures were physical TDV, sexual TDV, sexual violence by anyone (partner or nonpartner), bullied on school property, electronically bullied during the 12 months before the survey, and lifetime forced sexual intercourse. For each measure, dichotomous categories were created to indicate experiencing no victimization versus any victimization. The denominators for TDV victimization measures were students who reported dating during the 12 months before the survey; the denominators for sexual violence by anyone, lifetime forced sex, and bullying victimization measures were the full sample of students.

**TABLE 1:** Interpersonal violence victimization measures — Youth Risk Behavior Survey, United States, 2021

Health risk behavior	Questionnaire item	Analytic coding
**Violence victimization**		
Physical dating violence victimization	During the past 12 months, how many times did someone you were dating or going out with physically hurt you on purpose? (Count such things as being hit, slammed into something, or injured with an object or weapon.) [Excludes students who did not date or go out with anyone during the past 12 months] A. I did not date or go out with anyone during the past 12 months B. 0 times C. 1 time D. 2 or 3 times E. 4 or 5 times F. ≥6 times	>1 time versus 0 times
Sexual dating violence victimization	During the past 12 months, how many times did someone you were dating or going out with force you to do sexual things that you did not want to do? (Count such things as kissing, touching, or being physically forced to have sexual intercourse.) [Excludes students who did not date or go out with anyone during the past 12 months] A. I did not date or go out with anyone during the past 12 months B. 0 times C. 1 time D. 2 or 3 times E. 4 or 5 times F. ≥6 times	>1 time versus 0 times
Sexual violence victimization by anyone	During the past 12 months, how many times did anyone force you to do sexual things that you did not want to do? (Count such things as kissing, touching, or being physically forced to have sexual intercourse.) A. 0 times B. 1 time C. 2 or 3 times D. 4 or 5 times E. ≥6 times	>1 time versus 0 times
Bullied on school property	During the past 12 months, have you ever been bullied on school property? A. Yes B. No	Yes versus no
Electronically bullied	During the past 12 months, have you ever been electronically bullied? A. Yes B. No	Yes versus no
Forced sex	Have you ever been physically forced to have sexual intercourse when you did not want to? A. Yes B. No	Yes versus no

The two standard TDV victimization measures were combined into the following two composite measures: 1) experienced any TDV victimization (physical, sexual, or both) and 2) experienced both physical and sexual TDV victimization. Similarly, a bullying victimization “any” measure was created. The following student demographic characteristics were also included in analyses: sex (female and male); race and ethnicity (American Indian or Alaska Native [AI/AN], Asian, Black or African American [Black], Native Hawaiian or other Pacific Islander [NH/OPI], White, Hispanic or Latino [Hispanic], and multiracial); sexual identity (heterosexual, lesbian, gay, bisexual, questioning, or other); and sex of sexual contacts (opposite only, same only, or both sexes). (Persons of Hispanic origin might be of any race but are categorized as Hispanic; all racial groups are non-Hispanic.)

### Analysis

Prevalence for each form of IVV was estimated for all years with available data. To identify temporal trends, logistic regression analyses were used to model linear and quadratic time effects while controlling for sex, grade, and race and ethnicity changes over time. Time variables were treated as continuous and were coded by using orthogonal coefficients calculated with PROC IML in SAS (version 9.4; SAS Institute). Separate regression models were used to assess linear and quadratic trends for each variable; 3 years of survey data were required to calculate linear trends, and 6 survey years were required to calculate quadratic trends. Time effects with p values of <0.05 were considered statistically significant. When a statistically significant quadratic trend was identified, Joinpoint (version 4.9; National Cancer Institute) was used to identify the specific year where the change in trend occurred, and regression models were then used to identify linear trends occurring in each time segment. Significant differences in the 2-year prevalence of all the IVV measures (standard and composite) also were examined, using *t*-tests with Taylor series linearization to compare 2019 with 2021 (p<0.05).

Weighted prevalence estimates and corresponding 95% CIs were provided for all IVV measures. Comparisons by demographic characteristics and sex of sexual contacts were conducted using chi-square tests (p<0.05). When differences among subgroups were demonstrated, additional *t*-tests were performed to test pairwise differences between subpopulations. Differences between prevalence estimates were considered statistically significant if the *t*-test p-value was <0.05 for main effects (sex, race and ethnicity, sexual identity, and sex of sexual contacts). Analyses were completed using SAS (version 9.4; SAS Institute) and SUDAAN (version 11.0.3; RTI International) to account for the complex survey design and weighting.

## Results

Findings from the 2021 survey indicate that 8.5% of students who had dated in the past year experienced physical TDV and 9.7% experienced sexual TDV. Overall, 13.6% of students experienced any TDV (physical, sexual, or both), and 3.6% experienced both types of TDV (Table 2). In the full sample, 11.0% of students reported sexual violence victimization by anyone in the previous year. Of those students who reported sexual violence by anyone, 59.5% also reported sexual TDV. Lifetime forced sexual intercourse was reported by 8.5% of all students. Finally, 15.0% of students reported being bullied on school property, 15.9% reported electronic bullying, and 22.0% reported any bullying during the 12 months before the survey (Table 3).

**TABLE 2:** Prevalence of interpersonal violence victimization among high school students, by demographic characteristics and type of violence — Youth Risk Behavior Survey, United States, 2021*

Characteristic	Any TDV^†,§^		Physical TDV^†,¶^		Sexual TDV^†,^***	
	% (95% CI)	p value^††^	% (95% CI)	p value^††^	% (95% CI)	p value^††^
**Overall**	**13.6 (12.3–15.1)**	**NA**	**8.5 (7.6–9.6)**	**NA**	**9.7 (8.6–11.0)**	**NA**
**Sex**						
Female	19.0 (17.1–21.0)	<0.001	10.2 (8.9–11.6)	<0.001	15.3 (13.6–17.2)	<0.001
Male	8.2^§§^ (6.8–10.0)		6.7^§§^ (5.8–7.7)		4.0^§§^ (3.0–5.5)	
**Race and ethnicity^§§^**						
American Indian or Alaska Native	18.5 (8.6–35.4)	<0.001	14.6 (6.7–29.0)	0.003	11.0 (5.5–20.9)	<0.001
Asian	7.2***^,†††,§§§,¶¶¶^ (3.4–14.9)		5.3***^,¶¶¶^ (3.1–8.7)		5.5^†††^ (2.6–11.4)	
Black or African American	9.7***^,†††,¶¶¶^ (7.5–12.5)		8.1 (6.6–9.9)		5.3***^,¶¶¶^ (4.1–6.9)	
Native Hawaiian or other Pacific Islander	9.4^¶¶¶^ (4.0–20.4)		13.8 (5.6–30.4)		5.5 (1.1–23.9)	
White	14.9 (12.9–17.2)		9.1*** (7.9–10.5)		10.7 (8.8–13.0)	
Hispanic or Latino	13.2 (11.4–15.3)		7.4^¶¶^ (6.2–8.9)		10.0 (8.4–11.8)	
Multiracial	16.1 (11.9–21.5)		10.4 (7.5–14.1)		11.6 (8.3–16.1)	
**Sexual identity**						
Heterosexual (straight)	10.0****^,††††^ (8.9–11.2)	<0.001	6.2^††††^ (5.4–7.1)	<0.001	6.6^††††^ (5.6–7.7)	<0.001
Lesbian or gay	17.0^§§§^ (11.5–24.3)		14.1^§§§§^ (9.5–20.4)		12.0 (7.5–18.7)	
Bisexual	26.0^§§§^ (21.7–30.7)		16.1^§§§§^ (12.8–20.2)		20.8****^,§§§§^ (17.2–24.9)	
Questioning	21.1^§§§^ (15.4–28.1)		12.8^§§§§^ (8.7–18.5)		16.0^§§§§^ (11.7–21.6)	
Other	27.9^§§§^ (21.4–35.5)		16.5^§§§§^ (10.7–24.6)		23.8****^,§§§§^ (17.7–31.3)	
**Sex of sexual contacts**						
Opposite sex only	15.4 (13.6–17.4)	<0.001	9.3 (8.1–10.6)	<0.001	10.8 (9.2–12.6)	<0.001
Same sex only	16.4 (9.2–27.6)		12.6 (7.8–19.6)		11.2 (5.8–20.5)	
Both sexes	40.1^¶¶¶¶,^***** (34.6–45.9)		25.7^¶¶¶¶,^***** (19.5–33.2)		32.0^¶¶¶¶,^***** (26.8–37.7)	
**Total**	**Both physical and sexual TDV^†,†††††^**		**Sexual violence by anyone^§§§§§^**		**Forced sex (lifetime)**	
	**3.6 (2.9–4.5)**	**NA**	**11.0 (10.1–12.0)**	**NA**	**8.5 (7.6–9.4)**	**NA**
**Sex**						
Female	5.2 (4.2–6.5)	<0.001	17.9 (16.3–19.5)	<0.001	13.5 (12.3–14.8)	<0.001
Male	1.9^§§^ (1.3–2.7)		4.6^††^ (3.8–5.5)		3.6^††^ (2.8–4.4)	
**Race and ethnicity**						
American Indian or Alaska Native	5.9 (1.8–17.4)	0.779	15.8 (9.7–24.6)	<0.001	18.3 (12.1–26.6)	<0.001
Asian	3.1 (1.6–5.9)		5.7^¶¶,^***^,†††,§§§^ (3.4–9.4)		4.5^¶¶,^***^,†††,§§§^ (3.2–6.2)	
Black or African American	2.5 (1.4–4.5)		7.4^¶¶,^***^,†††,§§§^ (6.4–8.7)		7.1^†††,§§§^ (5.0–10.0)	
Native Hawaiian or other Pacific Islander	5.7 (1.0–27.2)		5.4^¶¶,^***^,†††,§§§^ (2.2–12.7)		9.8^†††^ (4.7–19.4)	
White	4.0 (2.8–5.5)		11.9 (10.7–13.3)		8.4^†††,§§§^ (7.4–9.5)	
Hispanic or Latino	3.7 (2.8–4.9)		11.3 (9.7–13.1)		9.5^†††^ (8.2–10.9)	
Multiracial	3.2 (2.0–5.1)		14.7 (11.3–18.7)		11.6 (9.1–14.5)	
**Sexual identity**						
Heterosexual (straight)	2.0 (1.5–2.5)	<0.001	7.6^††††^ (6.9–8.4)	<0.001	5.0^††††^ (4.3–5.8)	<0.001
Lesbian or gay	8.9^§§§§^ (5.1–15.1)		17.0^§§§§^ (12.7–22.5)		16.9^§§§§^ (12.7–22.2)	
Bisexual	9.6^††††,§§§§^ (6.6–13.7)		25.3****^,††††,§§§§^ (21.0–30.2)		23.3****^,††††,§§§§^ (20.5–26.3)	
Questioning	3.9 (1.8–8.2)		17.5^§§§§^ (14.6–20.8)		13.6^§§§§^ (10.9–16.9)	
Other	11.7^††††,§§§§^ (6.6–19.8)		20.9^§§§§^ (16.8–25.8)		19.0^††††,§§§§^ (14.6–24.4)	
**Sex of sexual contacts**						
Opposite sex only	3.6 (2.9–4.4)	<0.001	16.6 (15.0–18.4)	<0.001	12.7***** (11.1–14.5)	<0.001
Same sex only	4.3 (1.7–10.2)		25.5 (17.5–35.6)		22.7^¶¶¶¶^ (15.7–31.6)	
Both sexes	16.3^¶¶¶¶,^***** (11.1–23.4)		44.0^¶¶¶¶,^***** (39.2–49.0)		43.1^¶¶¶¶,^***** (38.7–47.6)	

**Abbreviations:** NA = not applicable; TDV = teen dating violence.

* N = 17,232 respondents. Because the state and local questionnaires differ by jurisdiction, students in these schools were not asked all national YRBS questions. Therefore, the total number (N) of students answering each question varied. Percentages in each category are calculated on the known data.

^†^ During the 12 months before the survey, among students who dated or went out with someone during the 12 months before the survey.

^§^ Combined any “yes” responses to physical TDV and sexual TDV.

^¶^ Being physically hurt on purpose (counting such things as being hit, slammed into something, or injured with an object or weapon) by someone they were dating or going out with, one or more times, among the 58.2% of students nationwide who dated or went out with someone during the 12 months before the survey.

** Being forced to do “sexual things” (counting such things as kissing, touching, or being physically forced to have sexual intercourse) they did not want to do by someone they were dating or going out with, one or more times, among the 58.0% of students nationwide who dated or went out with someone during the 12 months before the survey.

^†† ^p value is based on chi-square tests (p<0.05).

^§§^ Significantly different from female students, on the basis of *t*-test analysis with Taylor series linearization (p<0.05).

^¶¶ ^Persons of Hispanic or Latino (Hispanic) origin might be of any race but are categorized as Hispanic; all racial groups are non-Hispanic.

*** Significantly different from White students, based on *t*-test analysis with Taylor series linearization (p<0.05).

^†††^ Significantly different from Hispanic students, on the basis of *t*-test analysis with Taylor series linearization (p<0.05).

^§§§^ Significantly different from American Indian or Alaska Native students, on the basis of *t*-test analysis with Taylor series linearization (p<0.05).

^¶¶¶^ Significantly different from multiracial students, on the basis of *t*-test analysis with Taylor series linearization (p<0.05).

**** Significantly different from gay or lesbian students, on the basis of *t*-test analysis with Taylor series linearization (p<0.05).

^††††^ Significantly different from questioning students, on the basis of *t*-test analysis with Taylor series linearization (p<0.05).

^§§§§^ Significantly different from heterosexual students, on the basis of *t*-test analysis with Taylor series linearization (p<0.05).

^¶¶¶¶^ Significantly different from opposite sex only, on the basis of *t*-test analysis with Taylor series linearization (p<0.05).

***** Significantly different from same sex only, on the basis of *t*-test analysis with Taylor series linearization (p<0.05).

^†††††^ Combined where responses to both physical TDV and sexual TDV were “yes.”

^§§§§§^ Being forced to do “sexual things” (counting such things as kissing, touching, or being physically forced to have sexual intercourse) they did not want to do by anyone, one or more times during the 12 months before the survey.

**TABLE 3:** Prevalence of bullying victimization among high school students, by demographic characteristics and type of bullying — Youth Risk Behavior Survey, United States, 2021*

Characteristic	Any bullying^†,§^		Bullied on school property		Electronically bullied^†^	
	% (95% CI)	p value^¶^	% (95% CI)	p value^¶^	% (95% CI)	p value^¶^
**Overall**	**22.0 (21.0–23.0)**	**NA**	**15.0 (14.1–15.8)**	**NA**	**15.9 (15.0–16.8)**	**NA**
**Sex**						
Female	26.2 (24.9–27.6)	<0.001	17.0 (15.8–18.3)	<0.001	20.5 (19.3–21.7)	<0.001
Male	17.7** (16.0–19.5)		12.8** (11.3–14.5)		11.2** (10.3–12.2)	
**Race and ethnicity^††^**						
American Indian or Alaska Native	29.7 (21.9–39.0)	<0.001	17.8 (12.1–25.3)	<0.001	20.9 (14.3–29.4)	<0.001
Asian	17.3^§§,^***^,†††^ (13.3–22.3)		10.8^§§,†††^ (8.5–13.6)		13.3^§§^ (9.9–17.6)	
Black or African American	13.4^§§,¶¶,†††^ (11.7–15.2)		8.5^§§,¶¶,†††^ (7.2–10.1)		9.5^§§,¶¶,†††^ (8.4–10.9)	
Native Hawaiian or other Pacific Islander	14.0^§§,^*** (6.4–27.7)		8.9 (2.5–27.3)		9.7^§§,¶¶,^*** (5.9–15.5)	
White	26.3^††^ (24.6–28.1)		17.9^¶¶^ (16.6–19.3)		18.8^¶¶^ (17.2–20.4)	
Hispanic or Latino	17.9^§§,^*** (14.9–21.3)		12.4^§§^ (10.2–15.0)		13.2^§§,^*** (10.6–16.3)	
Multiracial	23.7 (19.1–28.9)		17.5 (13.3–22.8)		16.9 (12.8–22.0)	
**Sexual identity**						
Heterosexual (straight)	17.9^§§§^ (16.9–19.0)	<0.001	12.0 (11.1–12.9)	<0.001	12.7^¶¶¶^ (11.8–13.6)	<0.001
Lesbian or gay	35.2^¶¶¶,^**** (29.2–41.7)		26.4**** (21.4–32.2)		24.8**** (19.8–30.5)	
Bisexual	35.6^¶¶¶,^**** (32.5–38.9)		24.4**** (20.9–28.4)		28.4^¶¶¶,^**** (25.1–31.9)	
Questioning	26.4^§§§^ (23.3–29.7)		15.0 (12.0–18.5)		19.6**** (16.0–23.8)	
Other	41.0^¶¶¶,^**** (34.9–47.4)		29.3**** (23.9–35.4)		31.7^¶¶¶,^**** (26.4–37.5)	
**Sex of sexual contacts**						
Opposite sex only	24.8^††††^ (23.4–26.3)	<0.001	16.1^††††^ (14.8–17.4)	<0.001	19.5^††††^ (18.2–20.9)	<0.001
Same sex only	38.9^§§§^ (32.1–46.1)		29.3^§§§§^ (21.9–38.1)		31.8^§§§§^ (25.2–39.3)	
Both sexes	47.2^††††,§§§§^ (41.1–53.3)		33.1^§§§§^ (27.3–39.4)		39.1^§§§§^ (33.2–45.2)	

**Abbreviation:** NA = not applicable.

* N = 17,232 respondents. Because the state and local questionnaires differ by jurisdiction, students in these schools were not asked all national YRBS questions. Therefore, the total number (N) of students answering each question varied. Percentages in each category are calculated on the known data.

^†^ During the 12 months before the survey.

^§^ Combined any “yes” responses to bullied on school property and electronic bullying.

^¶^ p value is based on chi-square tests (p<0.05).

** Significantly different from female students, on the basis of *t*-test analysis with Taylor series linearization (p<0.05).

^††^ Persons of Hispanic or Latino (Hispanic) origin might be of any race but are categorized as Hispanic; all racial groups are non-Hispanic.

^§§^ Significantly different from White students, on the basis of *t*-test analysis with Taylor series linearization (p<0.05).

^¶¶^ Significantly different from Hispanic students, on the basis of *t*-test analysis with Taylor series linearization (p<0.05).

*** Significantly different from American Indian or Alaska Native students, on the basis of *t*-test analysis with Taylor series linearization (p<0.05).

^†††^ Significantly different from multiracial students, on the basis of *t*-test analysis with Taylor series linearization (p<0.05).

^§§§^ Significantly different from gay or lesbian students, on the basis of *t*-test analysis with Taylor series linearization (p<0.05).

^¶¶¶^ Significantly different from questioning students, on the basis of *t*-test analysis with Taylor series linearization (p<0.05).

**** Significantly different from heterosexual students, on the basis of *t*-test analysis with Taylor series linearization (p<0.05).

^††††^ Significantly different from same sex only, on the basis of *t*-test analysis with Taylor series linearization (p<0.05).

^§§§§^ Significantly different from opposite sex only, on the basis of *t*-test analysis with Taylor series linearization (p<0.05).

In 2021, differences for demographic characteristics and sex of sexual contacts were observed for the majority of IVV measures (Tables 2 and 3). Female students had greater prevalence of all types of IVV compared with male students. Variation in prevalence among racial and ethnic minority students was also observed for all types of IVV, although patterns were not consistent. AI/AN students reported the highest levels of TDV (including 18.5% prevalence of any TDV) and Asian, Black, and NH/OPI students reported the lowest levels. Differences were found in the prevalence of physical TDV for students who were multiracial (10.4%) or White (9.1%) compared with Asian students (5.3%), and Hispanic students (7.4%) had lower prevalence of physical TDV compared with White students (9.1%). Prevalence of sexual TDV was greater for students who were multiracial (11.6%), White (10.7%), or Hispanic (10.0%) compared with Black students (5.3%). The prevalence of sexual violence by anyone was greater for students who were AI/AN (15.8%), multiracial (14.7%), White (11.9%), or Hispanic (11.3%) compared with students who were Black (7.4%), Asian (5.7%), or NH/OPI (5.4%). Variation in the prevalence of lifetime forced sexual intercourse was observed for all racial and ethnic groups; however, the most consistent pattern was observed among AI/AN students, who had the greatest prevalence (18.3%) of forced sexual intercourse compared with students within all other racial and ethnic groups (range = 4.5%–9.8%) except multiracial students (11.6%). The patterns for being bullied on school property and electronic bullying were similar, with higher prevalence of bullying among AI/AN and White students and lower prevalence among Asian, Black, and NH/OPI students. Multiracial students had higher rates than Asian, Black, and NH/OPI students. AI/AN and White students tended to report any bullying at higher rates than Asian, Black, or Hispanic students.

The 2021 prevalence estimates for all forms of IVV also tended to be higher among students with a sexual identity other than heterosexual. Bisexual students had greater prevalence of sexual violence by anyone (25.3%) compared with those who identified as heterosexual (7.6%), lesbian or gay (17.0%), or questioning (17.5%). Students who identified as bisexual or other identity had greater prevalence of experiencing both types of TDV and any bullying than students who identified as questioning. Students who identified as lesbian or gay also reported any bullying at a greater prevalence than questioning students. All forms of TDV and sexual violence were reported at higher rates among students who reported sexual contact with both sexes than those who reported sexual contact with opposite sex only or same sex only. Students who reported sexual contact with both sexes or same sex only had a greater prevalence of being bullied on school property or electronically, and experiencing any bullying, than those with sexual contacts of the opposite sex only.

Trend analyses indicated that physical TDV, sexual TDV, experience of any TDV, and experiences with both physical and sexual TDV all decreased from 2013 to 2021 (Table 4). Being bullied on school property and any bullying victimization both decreased from 2011 to 2021. Lifetime forced sexual intercourse decreased during 2011–2015, then increased during 2015–2021. Sexual violence victimization by anyone increased during the period 2017–2021. Being bullied electronically did not change during the period 2011–2021; however, being bullied on school property decreased during 2017–2021 after being stable from 2011 to 2017. Few differences in types of IVV were observed from 2019 (pre–COVID-19 pandemic) to 2021 (during the COVID-19 pandemic). Being bullied on school property decreased from 19.5% to 15.0%, and the related composite measure (any bullying victimization) also decreased during this period from 24.8% to 22.0%. Sexual TDV increased from 8.2% in 2019 to 9.7% in 2021.

**TABLE 4:** Trends in prevalence of interpersonal violence victimization among high school students — Youth Risk Behavior Survey, United States, 2011–2021*

Interpersonal violence experience	Prevalence						Linear change^†^	Quadratic change^†^	Change during 2019–2021^§^
	2011	2013	2015	2017	2019	2021			
Any TDV^¶,^**	—^††^	15.7	15.6	11.7	12.2	13.6	Decreased	NA^§§^	No change
Physical TDV^¶^	—	10.3	9.6	8.0	8.2	8.5	Decreased	NA^§§^	No change
Sexual TDV^¶^	—	10.4	10.6	6.9	8.2	9.7	Decreased	NA^§§^	Increased
Both physical and sexual TDV^¶,¶¶^	—	4.9	4.6	2.5	3.0	3.6	Decreased	NA^§§^	No change
Sexual violence by anyone***	—	—	—	9.7	10.8	11.0	Increased	NA^§§^	No change
Forced sex (lifetime)	8.0	7.3	6.7	7.4	7.3	8.5	No linear change	Decreased 2011–2015 Increased 2015–2021	No change
**Bullying**									
Any bullying***^,†††^	27.0	25.2	25.7	24.0	24.8	22.0	Decreased	No quadratic change	Decreased
Bullied on school property***	20.1	19.6	20.2	19.0	19.5	15.0	Decreased	No linear change 2011–2017 Decreased 2017–2021	Decreased
Electronically bullied***	16.2	14.8	15.5	14.9	15.7	15.9	No linear change	No quadratic change	No change

**Abbreviations:** NA = not available; TDV = teen dating violence.

* N = 90,306 respondents. Because the state and local questionnaires differ by jurisdiction, students in these schools were not asked all national YRBS questions. Therefore, the total number (N) of students answering each question varied. Percentages in each category are calculated on the known data.

^†^ On the basis of trend analyses using a logistic regression model controlling for sex, race and ethnicity, and grade (p<0.05).

^§^ On the basis of *t*-test analysis with Taylor series linearization (p<0.05).

^¶^ During the 12 months before the survey, among students who dated or went out with someone during the 12 months before the survey.

** Combined any “yes” responses to physical TDV and sexual TDV.

^††^ Dashes indicate that no data are available.

^§§^ Insufficient years of data to assess quadratic trend.

^¶¶^ Combined where responses to both physical TDV and sexual TDV were “yes.”

*** During the 12 months before the survey.

^†††^ Combined any “yes” responses to bullied at school and electronically bullied.

## Discussion

This report describes 2021 prevalence estimates and trends in prevalence during 2011–2021 for different forms of IVV experienced by U.S. high school students. Findings indicate that multiple forms of TDV, sexual violence, and bullying victimization are common experiences for U.S. youths. Disparities in exposure also are evident, with female, racial and ethnic minority, and sexual minority youths disproportionately affected by these forms of violence in adolescence. Although other studies have demonstrated greater rates of violence among certain racial and ethnic and sexual minority groups (*1*,*2*), the number of subgroups examined with nationally representative data has been limited. This report presents data for additional population characteristics and behavior including AI/AN, Asian, NH/OPI, and multiracial youths; bisexual and questioning youths; and sex of sexual contacts, providing a nuanced context of prevalence and disparities among racial and ethnic and sexual minority youths.

Consistent with other studies, prevalence of both physical and sexual TDV was higher for females than males (*1*). Although males also report TDV victimization, factors including community norms that support gender inequity might increase the likelihood that females experience and report TDV (*4*). In addition, rates of both physical and sexual TDV were higher for AI/AN, NH/OPI, and multiracial youths than for White youths, and the prevalence of experiencing any TDV was highest for AI/AN youths. Trends indicate that sexual TDV increased from 2019 to 2021. Research has linked increases in stress and isolation to poor mental health in youths, which is associated with TDV (*5*). Although not yet examined, these effects might help explain this increase in sexual TDV during the pandemic period. The reasons why sexual TDV increased whereas physical TDV remained stable are unclear; additional research could examine whether factors such as technology-facilitated sexual violence (e.g., posting or sharing sexual pictures of someone without their consent, or nonconsensual sexting) and sexual harassment contribute to this finding. These trends and evidence of disparities in TDV experiences, with particularly vulnerable youths experiencing higher rates, highlight the need for comprehensive violence prevention efforts that are grounded in equity principles and address the unique needs of adolescents disproportionately affected by TDV.

Prevalence of lifetime forced sex and sexual violence victimization by anyone was higher for females than males, consistent with other studies (*1*). Rates of forced sex were also two to four times higher for AI/AN youths compared with other single-race groups, consistent with recent findings that nearly one in four AI/AN women experienced sexual abuse as a child, the highest rate among racial and ethnic groups (*6*). Of those students who reported sexual violence by anyone, 59.9% also reported sexual TDV, which indicates that a substantial portion of sexual violence victimization experiences were by someone other than a dating partner. Sexual violence in adolescence often is perpetrated by peers outside a dating context (*7*) and also can be perpetrated by family members, other known adults, and strangers, among others. Because of recent increases in lifetime forced sex (from 2015 to 2021) and past-year sexual violence victimization by anyone (from 2017 to 2021), prevention efforts that address sexual violence in both dating and nondating contexts are critical (https://www.cdc.gov/violenceprevention/pdf/2012FindingsonSVinYouth-508.pdf).

All forms of bullying victimization were more common among females, White youths, and sexual minority youths, consistent with previous research (*1*). In addition to White youths, AI/AN and multiracial youths had higher bullying rates than other racial and ethnic groups. Research on IVV experiences among AI/AN youths typically is limited to comparisons with White youths; therefore, these findings comparing AI/AN youths with other racial and ethnic minority youths provide needed data for the field (*8*,*9*). Tailoring prevention strategies to the cultural beliefs and norms of racial and ethnic minority subgroups that are disproportionately at risk for IVV might help address these disparities (*10*). Overall, rates of bullying victimization decreased from 2011 to 2021; however, the decrease in bullying on school property from 19.5% prepandemic (2019) to 15.0% during the COVID-19 pandemic (2021) was likely driven by reduced time spent on school property during 2020–2021. Electronic bullying rates remained stable, which is not a surprising finding because virtual learning and overall online interactions increased during the pandemic (*5*).

Sexual minority youths were at an increased risk for all forms of IVV included in this report compared with heterosexual youths. Although other studies indicate how sexual minority youths experience higher rates of bullying and sexual and physical violence compared with their peers who are not sexual minority youths, others excluded questioning youths and did not examine differences with bisexual youths or sex of sexual contacts (*2*,*11*). By disaggregating sexual minority youths and including identity and sex of sexual contacts (i.e., youths who identify as lesbian, gay, bisexual, questioning, or other and youths who have sexual contact with same-sex partners only and partners of both sexes), this report adds further context to national prevalence estimates of violence victimization against sexual minority youths; for example, students who identify as bisexual and students who have sexual contact with both sexes experience violence victimization at higher rates. School-based strategies to support LGBQ+ youths have been found to be associated with decreases in IVV among both LGBQ+ youths and heterosexual youths, contributing to safer school environments for all students (*12*). The consistent disparities in violence by sexual orientation found in this analysis highlight the important role of LGBTQ+ supportive practices in reducing experiences of violence.

Effective, evidence-based primary prevention is critical to reducing the substantial risk for violence victimization during high school, and research points to the importance of starting these prevention efforts early, before violence begins. Prevention strategies work best when they operate across levels of the social ecological model, addressing risk and protective factors of persons, their peers and families, and their physical and social environments (https://www.cdc.gov/violenceprevention/about/connectingthedots.html). CDC developed a series of guides that outline prevention resources to help communities identify effective approaches and implement comprehensive, multicomponent prevention efforts based on the best available research evidence to address sexual violence, youth violence, and intimate partner violence (https://www.cdc.gov/violenceprevention/communicationresources/pub/technical-packages.html#technicalPackages). For example, one prevention approach involves teaching youths how to act as engaged, proactive bystanders when they encounter sexist, homophobic, racist, or violence-supportive attitudes. Youth Voices in Prevention, a youth-led sexual violence prevention program, was found to increase bystander behaviors and decrease violence-related attitudes, with stronger effects for sexual minority and AI/AN youths (*13*). In addition, CDC developed Dating Matters: Strategies to Promote Healthy Teen Relationships, which includes prevention strategies focused on healthy relationship skills for youths and their families, schools, and neighborhoods.

Findings in this report highlight the importance of tailoring prevention strategies to create safe, nonjudgmental environments that promote protective factors to reduce disparities and increase safety among youths (*6*). Prevention efforts must also address disparities in risk for adolescent victimization by sex, race and ethnicity, and sexual minority status. Approaches should be designed or adapted to address the unique social and structural risk and protective factors affecting these groups, including social determinants of health (e.g., racism, discrimination, and socioeconomic disadvantage) that perpetuate and reinforce health disparities (*14*,*15*). For example, approaches that strengthen household financial security, create safer and healthier communities through physical environment enhancements, or connect youths to caring adults through mentoring or job training programs can help build protective environments for youths at higher risk for violence exposure (https://www.cdc.gov/violenceprevention/pdf/yv-technicalpackage.pdf) (*14*).

## Limitations

General limitations for the YRBS are available in the overview report of this supplement (*3*). The findings in this report are subject to at least four additional limitations. First, because of the breadth of topics included in the YRBS, the violence subtype measures included in the YRBS, and in this report, were assessed by single items, which might not capture all the dimensions of a construct. Second, substantial overlap likely existed in the measures that examined experiences of sexual violence victimization (i.e., sexual dating violence victimization and sexual violence victimization by anyone) and among the bullying victimization measures (i.e., electronic bullying and bullied at school). For these reasons, composites for the sexual violence measures and a “both” composite for bullying (i.e., experienced both electronic bullying and bullying at school) were not created. Third, the forms of violence assessed in this report do not encompass the full range of violence experiences in adolescence, and patterns of victimization across groups (e.g., by sex or race and ethnicity) for other types of violence might be different from those identified for TDV, sexual violence, and bullying in this report. Finally, the sexual violence measures had higher levels of missingness than other outcomes in this report (17.8%, forced sex; 22.6% sexual violence by anyone; and 24.5%, sexual TDV among the 17,232 respondents) attributed, at least in part, to the use of different versions of the YRBS questionnaire in specific states and localities that did not include sexual violence questions. More information on missingness of YRBS data is available in the overview report of this supplement (*3*). Although the proportion of missing data for sexual violence questions is consistent with previous YRBS cycles, prevalence estimates for sexual violence measures might be over- or underestimated.

## Future Directions

Identifying the differential burden of adolescent IVV among the demographic groups included in this report is important. Although this IVV report disaggregated racial and ethnic groups as much as was feasible with these 2021 YRBS data, each group presented is not homogenous. Victimization studies that further disaggregate the categories of racial and ethnic groups and also explore the intersection of race and ethnicity, sex, and sexual identity, might add additional information that can be used to tailor prevention and intervention efforts for those populations. Future research on IVV that includes transgender youths would advance our ability to understand the needs of transgender youths; the 2023 YRBS will measure transgender identity (https://www.cdc.gov/healthyyouth/data/yrbs/pdf/2023/2023_yrbs_national_hs_questionnaire.pdf). In addition, national estimates of other forms of violence victimization in adolescence, such as physical assault and homicide, are needed to provide a broader picture of violence risks for youths, including violence that might disproportionately affect males (https://www.cdc.gov/violenceprevention/communityviolence/index.html).

## Conclusion

Violence victimization among youths is a public health concern because experiences of IVV during childhood have been associated with increased risk for adverse experiences and poor health outcomes during adulthood (*1*). This report used nationally representative data from the 2021 YRBS to estimate the prevalence of TDV, sexual violence, and bullying victimization among U.S. high school students by demographic characteristics and sex of sexual contacts. Understanding disparities in IVV can be useful in prevention efforts for youths who are disproportionately affected by violence. Prevention approaches that focus not just on the personal, family, or school level but also incorporate an understanding of the social determinants of health (*15*) might be more effective for reducing violence experienced by youths among disproportionately affected populations.

## Footnotes

* See e.g., 45 C.F.R. part 46.102(l)(2), 21 C.F.R. part 56; 42 U.S.C. §241(d); 5 U.S.C. §552a; 44 U.S.C. §3501 et seq.
